# Crystal structures of *rac*-2,3-diphenyl-2,3,5,6-tetra­hydro-4*H*-1,3-thia­zine-1,1,4-trione and *N*-[(2*S*,5*R*)-1,1,4-trioxo-2,3-diphenyl-1,3-thia­zinan-5-yl]acet­amide

**DOI:** 10.1107/S2056989023000695

**Published:** 2023-01-31

**Authors:** Hemant P. Yennawar, Saige L. Lowe, Matthew M. Mammen, Connor R. Verhagen, Lee J. Silverberg

**Affiliations:** a Department of Biochemistry and Molecular Biology, Pennsylvania State University, University Park, PA 16802, USA; b Pennsylvania State University, Schuylkill Campus, 200 University Drive, Schuylkill Haven, PA 17972, USA; University of Aberdeen, United Kingdom

**Keywords:** crystal structure, thia­zinone, sulfone, enanti­opure

## Abstract

The syntheses and crystal structures of two thia­zinone compounds, namely, *rac*-2,3-diphenyl-2,3,5,6-tetra­hydro-4*H*-1,3-thia­zine-1,1,4-trione, C_16_H_15_NO_3_S, in its racemic form, and *N*-[(2*S*,5*R*)-1,1,4-trioxo-2,3-diphenyl-1,3-thia­zinan-5-yl]acet­amide, C_18_H_18_N_2_O_4_S, in an enanti­opure form, are reported. The thia­zine rings in the two structures differ in their puckering, as a half-chair in the first and a boat pucker in the second. The extended structures for both compounds have only C—H⋯O-type inter­actions between symmetry-related mol­ecules, and exhibit no π–π stacking inter­actions in spite of each having two phenyl rings.

## Abstract 2

The syntheses and crystal structures of two thia­zinone compounds, in an enanti­opure form, are reported. The thia­zine rings in the two structures differ in their puckering, as a half-chair in the first and a boat pucker in the second.

## 1. Chemical context

The 2,3-di­hydro-4*H*-1,3-thia­zin-4-ones are a group of six-membered heterocycles with a wide range of biological activity (Ryabukhin *et al.*, 1996 ▸; Silverberg & Moyer, 2019 ▸). Surrey’s research (Surrey *et al.*, 1958 ▸; Surrey, 1963*a* ▸,*b* ▸) resulted in the discovery of two drugs, the anti­anxiety and muscle relaxant chlormezanone, C_11_H_12_ClNO_3_S, [2-(4-chloro­phen­yl)-3-methyl-2,3,5,6-tetra­hydro-4*H*-1,3-thia­zin-4-one 1,1-dioxide] (O’Neil, 2006 ▸; Tanaka & Horayama, 2005 ▸) and the muscle relaxant dichlormezanone, C_11_H_11_Cl_2_NO_3_S, [2-(3,4-di­chloro­phen­yl)-3-methyl-2,3,5,6-tetra­hydro-4*H*-1,3-thia­zin-4-one 1,1-dioxide] (Elks & Ganellin, 1990 ▸). These sulfones showed greater activity than the sulfides from which they were synthesized (Surrey *et al.*, 1958 ▸). 
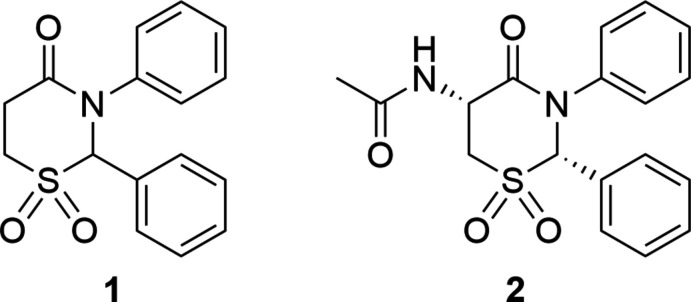


We have previously reported the preparation of the sulfones *rac*-2,3-di­hydro-2,3-diphenyl-4*H*-1,3-thia­zin-4-one 1,1-dioxide and *N*-[(2*S*,5*R*)-1,1-dioxido-4-oxo-2,3-diphenyl-1,3-thia­zinan-5-yl]acetamide (Silverberg, 2020 ▸). We have also reported X-ray crystal structures of the corresponding sulfides and sulfoxides (Yennawar & Silverberg, 2014 ▸, 2015 ▸; Yennawar *et al.*, 2015 ▸, 2016 ▸, 2017 ▸). The crystal structure of chlormezanone has been reported (Tanaka & Horayama, 2005 ▸). Herein we report the crystal structures of *rac*-2,3-diphenyl-2,3,5,6-tetra­hydro-4*H*-1,3-thia­zine-1,1,4-trione, **1**, and *N*-[(2*S*,5*R*)-1,1,4-trioxo-2,3-diphenyl-1,3-thia­zinan-5-yl]acet­amide, **2**.

## 2. Structural commentary

Compound **1** has one chiral center at C1 with an *S* configuration in the arbitrarily chosen asymmetric unit but crystal symmetry generates a racemic mixture (space group *P*2_1_/*c*). Compound **2** has two chiral centers, at C1 and C3 (*S* and *R* respectively), synthesized as such, and crystallizes in space group *P*2_1_2_1_2_1_. In **1**, the dihedral angles between the thia­zine ring (all atoms) and the pendant C5–C10 and C11–C16 phenyl groups are 84.02 (14) and 79.56 (12)°, respectively; the dihedral angle between the pendant rings is 61.26 (15)°. The equivalent angles in **2** are 81.25 (15), 82.58 (13) and 50.40 (15)°, respectively.

**Figure 1:**
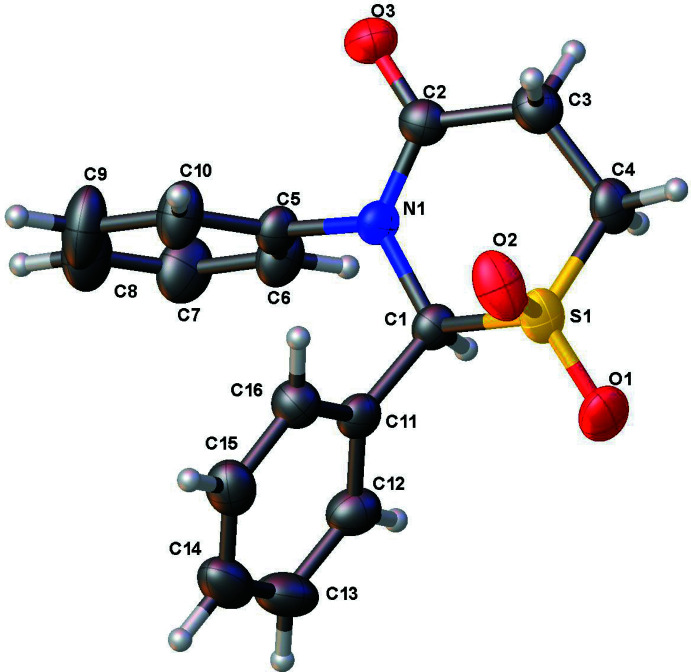
The asymmetric unit of **1** with displacement ellipsoids drawn at 50% probability level.

**Figure 2:**
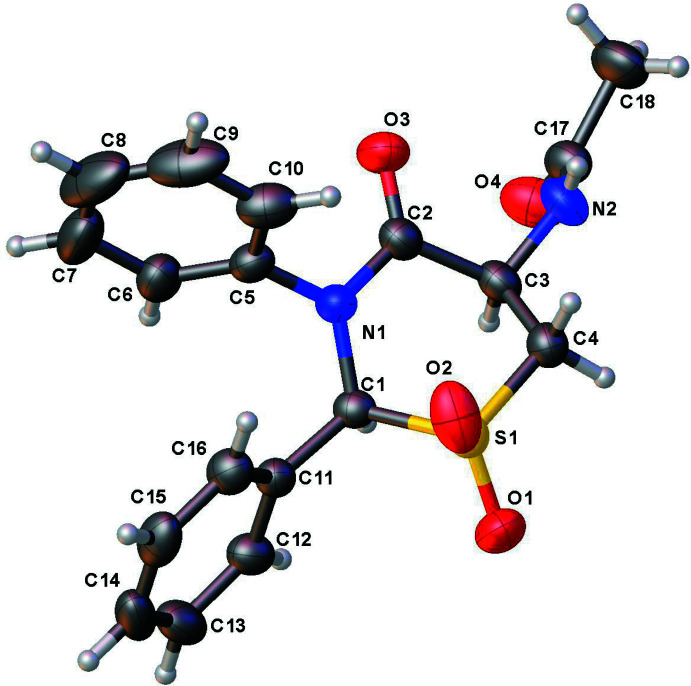
The asymmetric unit of **2** with displacement ellipsoids drawn at 50% probability level.

**Figure 3:**
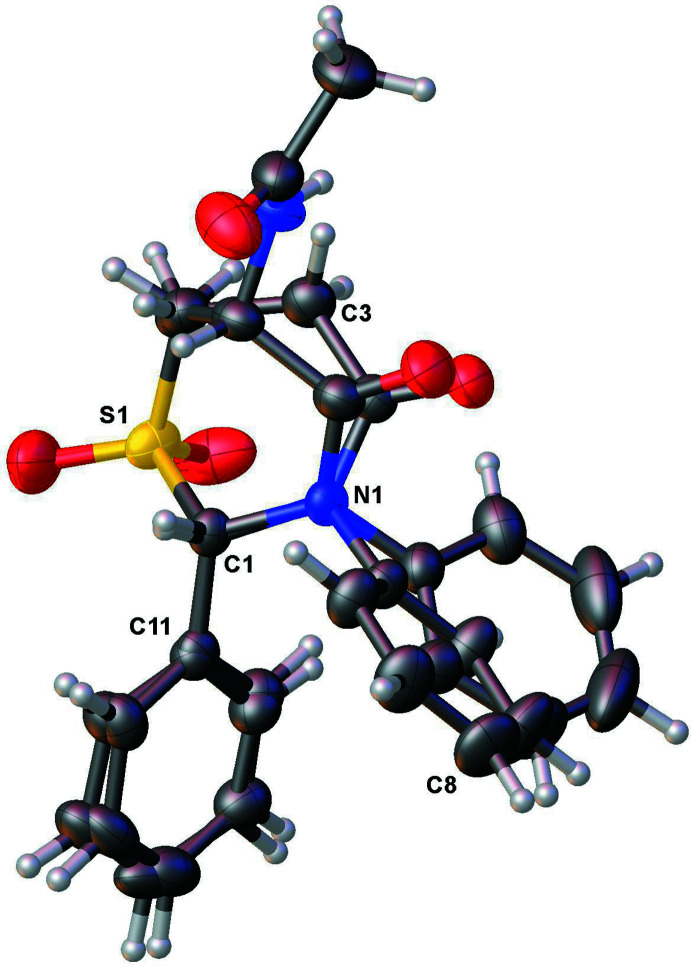
Overlay plot of **1** and **2** where the three atoms S1, N1, and C11 are matched. Atoms C3 and C8 of compound **1** are labeled.

The structure of **1** (Fig. 1 ▸) has a half-chair puckering of the thia­zine ring with puckering amplitude *Q* = 0.605 (2) Å, θ = 47.2 (2)°, φ = 346.7 (3)°, while in **2** (Fig. 2 ▸) the ring has a boat pucker [*Q* = 0.770 (2) Å, θ = 85.31 (15)°, φ = 61.89 (17)°]. This change in the puckering of the central ring system of the two mol­ecules leads to differing orientations of one of the phenyl rings, which is clear from the overlay diagram (Fig. 3 ▸).

## 3. Supra­molecular features

**Figure 4:**
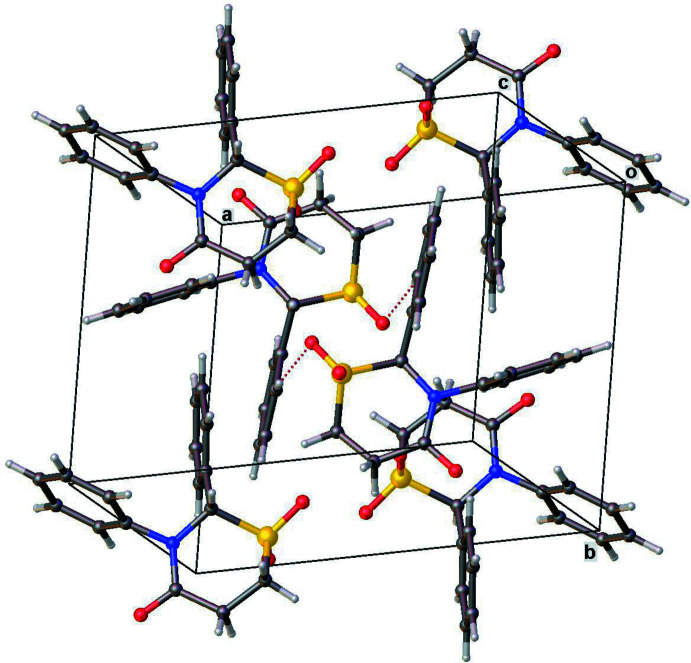
Crystal packing diagram for **1** showing inter­molecular pairs of C—H⋯O hydrogen bonds.

**Figure 5:**
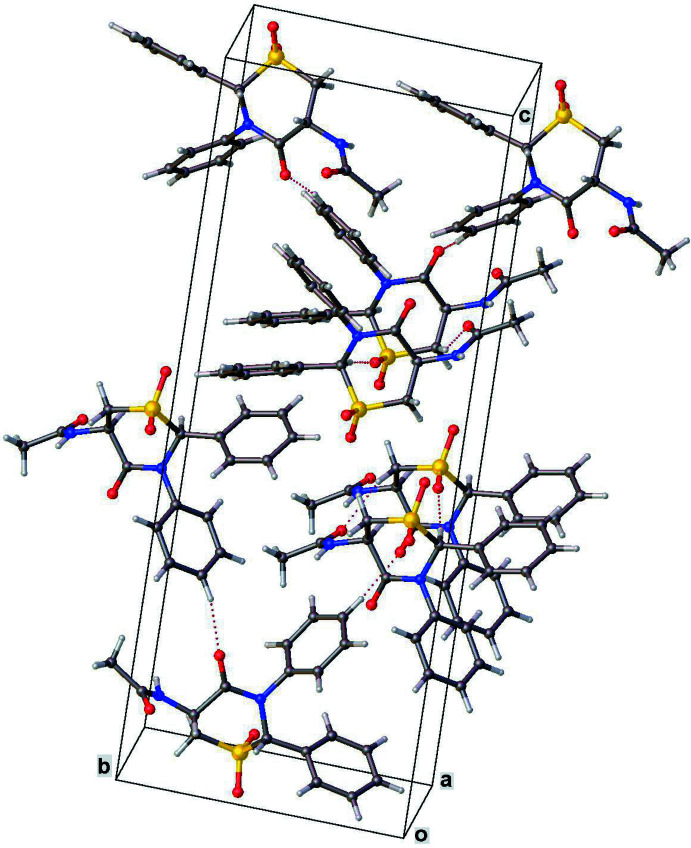
Crystal packing diagram for **2** showing inter­molecular C—H⋯O hydrogen bonds.

In both structures, only C—H⋯O-type hydrogen-bond inter­actions between symmetry-related mol­ecules are observed (Tables 1 ▸ and 2 ▸). In **1**, a single hydrogen bond [C12—H12⋯O1 = 3.454 (4) Å, 157°] and its symmetry-equivalent form a pair of parallel inter­actions (Fig. 4 ▸). In **2** (Fig. 5 ▸), the carbon atoms C1 and C4, both of the thia­zine ring, as well as C8 of one of the phenyl rings each donate an H atom for three distinct inter­actions involving three of the four oxygen atoms in the mol­ecule. Although both compounds each have two phenyl rings, neither of the lattices exhibit any π–π stacking inter­actions.

## 4. Database survey

Searches undertaken using the American Chemical Society’s Chemical Abstract Service (CAS) Scifinder platform did not find crystal structures of any 1,3-thia­zin-4-one sulfones other than chlormezanone (CSD refcode KAPNAR; Tanaka & Horayama, 2005 ▸).

## 5. Synthesis and crystallization

**General oxidation procedure** (Surrey *et al.*, 1958 ▸; Silverberg, 2020 ▸; Cannon *et al.* 2015 ▸): the heterocycle (0.267 mmol) was dissolved in glacial acetic acid (1.2 ml). An aqueous solution of KMnO_4_ (0.535 mmol in 1.45 ml water) was added dropwise at room temperature with vigorous stirring. The reaction was followed by TLC. Solid sodium bis­ulfite (NaHSO_3_/Na_2_S_2_O_5_) was added until the mixture remained colorless and then 1.45 ml of water were added and stirred for 10 min. The mixture was extracted with CH_2_Cl_2_ (3 × 5 ml). The organics were combined and washed once with sat. NaCl. The solution was dried over Na_2_SO_4_ and filtered. The product was purified by chromatography in a silica gel micro-column.

***rac*-2,3-diphenyl-2,3,5,6-tetra­hydro-4*H*-1,3-thia­zine-1,1,4-trione, 1:** Eluted with mixtures of ethyl acetate and hexa­nes. White solid (0.053 g, 70%). m.p.: 418–421 K. Crystals for X-ray diffraction studies were grown by slow evaporation from toluene solution.

***N*-[(2*S*,5*R*)-1,1,4-trioxo-2,3-diphenyl-1,3-thia­zinan-5-yl]acet­amide, 2:** Eluted with a mixture of 10% acetone and 90% ethyl acetate. White solid (0.076 g, 80%). m.p.: 443–467 K (decomposition). Crystals were grown by slow evaporation from ethanol solution.

## 6. Refinement

Crystal data, data collection and structure refinement details are summarized in Table 3 ▸. The hydrogen atoms were placed in their geometrically calculated positions and their coordin­ates refined using the riding model with parent-atom—H lengths of 0.93 Å (CH), 0.98 Å (chiral-CH), 0.96 Å (CH_3_), 0.97 Å (CH_2_). Isotropic displacement parameters for these atoms were set to 1.2 (CH) or 1.5 (CH_3_) times *U*_eq_ of the parent atom.

## Supplementary Material

Crystal structure: contains datablock(s) 1, 2. DOI: 10.1107/S2056989023000695/hb8050sup1.cif

Structure factors: contains datablock(s) 1. DOI: 10.1107/S2056989023000695/hb80501sup2.hkl

Supporting information file. DOI: 10.1107/S2056989023000695/hb80501sup4.mol

Structure factors: contains datablock(s) 2. DOI: 10.1107/S2056989023000695/hb80502sup3.hkl

Supporting information file. DOI: 10.1107/S2056989023000695/hb80502sup5.mol

Supporting information file. DOI: 10.1107/S2056989023000695/hb80501sup6.cml

Supporting information file. DOI: 10.1107/S2056989023000695/hb80502sup7.cml

CCDC references: 2238010, 2238009

Additional supporting information:  crystallographic information; 3D view; checkCIF report

## Figures and Tables

**Table 1:** Hydrogen-bond geometry (Å, °) for **1** [Chem scheme1]

*D*—H⋯*A*	*D*—H	H⋯*A*	*D*⋯*A*	*D*—H⋯*A*
C12—H12⋯O1^i^	0.95	2.56	3.454 (4)	157

Symmetry code: (i) 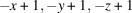 .

**Table 2:** Hydrogen-bond geometry (Å, °) for **2** [Chem scheme1]

*D*—H⋯*A*	*D*—H	H⋯*A*	*D*⋯*A*	*D*—H⋯*A*
C1—H1⋯O2^i^	0.98	2.39	3.365 (3)	173
C4—H4*A*⋯O4^ii^	0.97	2.29	3.185 (4)	153
C8—H8⋯O3^iii^	0.93	2.51	3.378 (5)	155

Symmetry codes: (i) 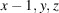 ; (ii) 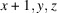 ; (iii) 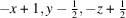 .

**Table 3:** Experimental details

	**1**	**2**
Crystal data		
Chemical formula	C_16_H_15_NO_3_S	C_18_H_18_N_2_O_4_S
*M*_r_	301.35	358.40
Crystal system, space group	Monoclinic, *P*2_1_/*c*	Orthorhombic, *P*2_1_2_1_2_1_
Temperature (K)	173	298
*a*, *b*, *c* (Å)	14.4485 (6), 10.2031 (5), 10.4950 (4)	5.5230 (4), 10.6857 (9), 28.430 (2)
α, β, γ (°)	90, 107.179 (4), 90	90, 90, 90
*V* (Å^3^)	1478.13 (11)	1677.8 (2)
*Z*	4	4
Radiation type	Cu *K*α	Mo *K*α
μ (mm^−1^)	2.03	0.22
Crystal size (mm)	0.2 × 0.18 × 0.09	0.22 × 0.06 × 0.06

Data collection		
Diffractometer	Rigaku Oxford Diffraction Synergy Custom system, HyPix-Arc 150	Bruker SMART CCD area detector
Absorption correction	Multi-scan (*CrysAlis PRO*; Rigaku OD, 2022 ▸)	Multi-scan (*SADABS*; Krause *et al.*, 2015 ▸)
*T*_min_, *T*_max_	0.668, 1.000	0.656, 0.900
No. of measured, independent and observed [*I* > 2σ(*I*)] reflections	7437, 2856, 2139	13301, 4037, 3460
*R*_int_	0.056	0.035
(sin θ/λ)_max_ (Å^−1^)	0.628	0.667

Refinement		
*R*[*F* ^2^ > 2σ(*F* ^2^)], *wR*(*F* ^2^), *S*	0.052, 0.150, 1.10	0.042, 0.103, 1.04
No. of reflections	2856	4037
No. of parameters	191	231
H-atom treatment	H-atom parameters constrained	H atoms treated by a mixture of independent and constrained refinement
Δρ_max_, Δρ_min_ (e Å^−3^)	0.32, −0.34	0.24, −0.14
Absolute structure	–	Flack *x* determined using 1213 quotients [(*I* ^+^)−(*I* ^−^)]/[(*I* ^+^)+(*I* ^−^)] (Parsons *et al.*, 2013 ▸)
Absolute structure parameter	–	0.07 (4)

Computer programs: *CrysAlis PRO* (Rigaku OD, 2022 ▸), *SMART* and *SAINT* (Bruker, 2016 ▸), *SHELXT* (Sheldrick, 2015*a* ▸), *SHELXS* (Sheldrick, 2008 ▸), *SHELXL2018/3* (Sheldrick, 2015*b* ▸) and *OLEX2* (Dolomanov *et al.*, 2009 ▸).

## supplementary crystallographic information

### rac-2,3-Diphenyl-2,3,5,6-tetrahydro-4H-1,3-thiazine-1,1,4-trione (1) Crystal data

**Table:**

C_16_H_15_NO_3_S	*F*(000) = 632
*M**_r_* = 301.35	*D*_x_ = 1.354 Mg m^−^^3^
Monoclinic, *P*2_1_/*c*	Cu *K*α radiation, λ = 1.54184 Å
*a* = 14.4485 (6) Å	Cell parameters from 3416 reflections
*b* = 10.2031 (5) Å	θ = 3.2–73.3°
*c* = 10.4950 (4) Å	µ = 2.03 mm^−^^1^
β = 107.179 (4)°	*T* = 173 K
*V* = 1478.13 (11) Å^3^	Block, clear colourless
*Z* = 4	0.2 × 0.18 × 0.09 mm

### rac-2,3-Diphenyl-2,3,5,6-tetrahydro-4H-1,3-thiazine-1,1,4-trione (1) Data collection

**Table:**

Rigaku Oxford Diffraction Synergy Custom system, HyPix-Arc 150 diffractometer	2856 independent reflections
Radiation source: Rotating-anode X-ray tube, Rigaku (Cu) X-ray Source	2139 reflections with *I* > 2σ(*I*)
Mirror monochromator	*R*_int_ = 0.056
Detector resolution: 10.0000 pixels mm^-1^	θ_max_ = 75.6°, θ_min_ = 3.2°
ω scans	*h* = −15→17
Absorption correction: multi-scan (CrysAlisPro; Rigaku OD, 2022)	*k* = −11→12
*T*_min_ = 0.668, *T*_max_ = 1.000	*l* = −12→13
7437 measured reflections	

### rac-2,3-Diphenyl-2,3,5,6-tetrahydro-4H-1,3-thiazine-1,1,4-trione (1) Refinement

**Table:**

Refinement on *F*^2^	Hydrogen site location: inferred from neighbouring sites
Least-squares matrix: full	H-atom parameters constrained
*R*[*F*^2^ > 2σ(*F*^2^)] = 0.052	*w* = 1/[σ^2^(*F*_o_^2^) + (0.0712*P*)^2^ + 0.268*P*] where *P* = (*F*_o_^2^ + 2*F*_c_^2^)/3
*wR*(*F*^2^) = 0.150	(Δ/σ)_max_ < 0.001
*S* = 1.10	Δρ_max_ = 0.32 e Å^−^^3^
2856 reflections	Δρ_min_ = −0.34 e Å^−^^3^
191 parameters	Extinction correction: *SHELXL2018/3* (Sheldrick 2015b), Fc^*^=kFc[1+0.001xFc^2^λ^3^/sin(2θ)]^-1/4^
0 restraints	Extinction coefficient: 0.0023 (5)
Primary atom site location: dual	

### rac-2,3-Diphenyl-2,3,5,6-tetrahydro-4H-1,3-thiazine-1,1,4-trione (1) Special details

**Table:**

Geometry. All esds (except the esd in the dihedral angle between two l.s. planes) are estimated using the full covariance matrix. The cell esds are taken into account individually in the estimation of esds in distances, angles and torsion angles; correlations between esds in cell parameters are only used when they are defined by crystal symmetry. An approximate (isotropic) treatment of cell esds is used for estimating esds involving l.s. planes.

### rac-2,3-Diphenyl-2,3,5,6-tetrahydro-4H-1,3-thiazine-1,1,4-trione (1) Fractional atomic coordinates and isotropic or equivalent isotropic displacement parameters (Å^2^)

**Table:**

	*x*	*y*	*z*	*U*_iso_*/*U*_eq_	
S1	0.45082 (4)	0.66477 (7)	0.66401 (5)	0.0380 (2)	
O1	0.52700 (12)	0.5704 (2)	0.67907 (19)	0.0514 (6)	
O2	0.41925 (14)	0.6961 (2)	0.77812 (16)	0.0519 (6)	
O3	0.22170 (13)	0.91599 (19)	0.44575 (18)	0.0455 (5)	
N1	0.27444 (13)	0.7076 (2)	0.48536 (18)	0.0306 (5)	
C1	0.35000 (16)	0.6063 (3)	0.5290 (2)	0.0310 (5)	
H1	0.374440	0.583984	0.451921	0.037*	
C4	0.47674 (18)	0.8100 (3)	0.5937 (2)	0.0396 (6)	
H4A	0.532525	0.854461	0.656910	0.048*	
H4B	0.493981	0.790381	0.511127	0.048*	
C3	0.38813 (19)	0.8988 (3)	0.5617 (3)	0.0425 (6)	
H3A	0.399282	0.971486	0.505438	0.051*	
H3B	0.384838	0.938047	0.646531	0.051*	
C2	0.28912 (18)	0.8402 (3)	0.4920 (2)	0.0348 (6)	
C5	0.18322 (17)	0.6594 (3)	0.3978 (2)	0.0333 (6)	
C6	0.17961 (19)	0.6182 (3)	0.2711 (2)	0.0440 (7)	
H6	0.235336	0.625452	0.241121	0.053*	
C7	0.0947 (2)	0.5664 (4)	0.1879 (3)	0.0559 (9)	
H7	0.091908	0.537900	0.100655	0.067*	
C8	0.0141 (2)	0.5563 (4)	0.2324 (3)	0.0633 (10)	
H8	−0.044126	0.519958	0.175939	0.076*	
C9	0.0179 (2)	0.5990 (4)	0.3588 (3)	0.0682 (11)	
H9	−0.038042	0.592964	0.388363	0.082*	
C10	0.10296 (19)	0.6506 (3)	0.4427 (3)	0.0496 (8)	
H10	0.105807	0.679455	0.529797	0.060*	
C11	0.31292 (16)	0.4818 (3)	0.5756 (2)	0.0334 (6)	
C12	0.3255 (2)	0.3631 (3)	0.5188 (3)	0.0437 (7)	
H12	0.356067	0.360439	0.450153	0.052*	
C13	0.2933 (2)	0.2481 (3)	0.5624 (3)	0.0550 (8)	
H13	0.301505	0.166705	0.523015	0.066*	
C14	0.2495 (2)	0.2515 (3)	0.6624 (3)	0.0541 (8)	
H14	0.227909	0.172599	0.692365	0.065*	
C15	0.2371 (2)	0.3693 (3)	0.7189 (3)	0.0468 (7)	
H15	0.207209	0.371115	0.788272	0.056*	
C16	0.26749 (17)	0.4849 (3)	0.6758 (2)	0.0391 (6)	
H16	0.257539	0.566039	0.714104	0.047*	

### rac-2,3-Diphenyl-2,3,5,6-tetrahydro-4H-1,3-thiazine-1,1,4-trione (1) Atomic displacement parameters (Å^2^)

**Table:**

	*U*^11^	*U*^22^	*U*^33^	*U*^12^	*U*^13^	*U*^23^
S1	0.0329 (4)	0.0492 (5)	0.0252 (3)	−0.0062 (3)	−0.0018 (2)	0.0054 (3)
O1	0.0332 (10)	0.0563 (14)	0.0540 (11)	0.0048 (9)	−0.0038 (8)	0.0172 (10)
O2	0.0568 (12)	0.0699 (15)	0.0249 (9)	−0.0242 (11)	0.0060 (8)	−0.0045 (9)
O3	0.0431 (11)	0.0385 (11)	0.0531 (11)	0.0069 (9)	0.0117 (8)	0.0068 (9)
N1	0.0264 (10)	0.0339 (12)	0.0271 (9)	0.0014 (9)	0.0010 (7)	0.0010 (9)
C1	0.0288 (12)	0.0369 (14)	0.0242 (11)	0.0019 (11)	0.0029 (8)	0.0001 (10)
C4	0.0332 (13)	0.0488 (18)	0.0306 (12)	−0.0078 (12)	−0.0002 (9)	−0.0007 (12)
C3	0.0429 (15)	0.0410 (16)	0.0409 (13)	−0.0035 (13)	0.0085 (11)	−0.0046 (12)
C2	0.0387 (13)	0.0361 (15)	0.0287 (12)	0.0004 (12)	0.0088 (9)	0.0008 (11)
C5	0.0266 (12)	0.0412 (16)	0.0273 (11)	0.0008 (11)	0.0006 (9)	0.0025 (11)
C6	0.0344 (13)	0.061 (2)	0.0318 (13)	0.0004 (13)	0.0024 (10)	−0.0038 (13)
C7	0.0457 (16)	0.075 (2)	0.0375 (14)	−0.0016 (16)	−0.0020 (11)	−0.0111 (15)
C8	0.0393 (16)	0.083 (3)	0.0542 (18)	−0.0118 (17)	−0.0070 (13)	−0.0056 (18)
C9	0.0319 (15)	0.118 (3)	0.0511 (18)	−0.0105 (19)	0.0072 (12)	0.003 (2)
C10	0.0347 (14)	0.079 (2)	0.0340 (13)	−0.0036 (15)	0.0083 (10)	−0.0019 (14)
C11	0.0284 (12)	0.0365 (15)	0.0299 (11)	0.0023 (11)	0.0005 (9)	0.0008 (11)
C12	0.0441 (15)	0.0421 (17)	0.0402 (14)	0.0065 (13)	0.0051 (11)	−0.0036 (13)
C13	0.063 (2)	0.0324 (17)	0.0579 (18)	0.0028 (15)	0.0007 (15)	0.0000 (14)
C14	0.0525 (17)	0.0420 (19)	0.0573 (18)	−0.0064 (15)	−0.0001 (14)	0.0102 (15)
C15	0.0428 (15)	0.0516 (19)	0.0431 (15)	−0.0038 (14)	0.0083 (11)	0.0098 (14)
C16	0.0398 (14)	0.0400 (16)	0.0360 (13)	−0.0006 (12)	0.0089 (10)	0.0013 (12)

### rac-2,3-Diphenyl-2,3,5,6-tetrahydro-4H-1,3-thiazine-1,1,4-trione (1) Geometric parameters (Å, º)

**Table:**

S1—O1	1.435 (2)	C6—C7	1.383 (4)
S1—O2	1.438 (2)	C7—H7	0.9500
S1—C1	1.807 (2)	C7—C8	1.381 (4)
S1—C4	1.744 (3)	C8—H8	0.9500
O3—C2	1.226 (3)	C8—C9	1.382 (5)
N1—C1	1.475 (3)	C9—H9	0.9500
N1—C2	1.368 (3)	C9—C10	1.387 (4)
N1—C5	1.451 (3)	C10—H10	0.9500
C1—H1	1.0000	C11—C12	1.385 (4)
C1—C11	1.514 (4)	C11—C16	1.394 (3)
C4—H4A	0.9900	C12—H12	0.9500
C4—H4B	0.9900	C12—C13	1.389 (4)
C4—C3	1.523 (4)	C13—H13	0.9500
C3—H3A	0.9900	C13—C14	1.377 (5)
C3—H3B	0.9900	C14—H14	0.9500
C3—C2	1.524 (4)	C14—C15	1.375 (4)
C5—C6	1.381 (3)	C15—H15	0.9500
C5—C10	1.377 (4)	C15—C16	1.381 (4)
C6—H6	0.9500	C16—H16	0.9500
			
O1—S1—O2	118.62 (12)	C5—C6—H6	120.0
O1—S1—C1	106.20 (12)	C5—C6—C7	119.9 (3)
O1—S1—C4	111.28 (13)	C7—C6—H6	120.0
O2—S1—C1	110.23 (11)	C6—C7—H7	120.2
O2—S1—C4	108.92 (14)	C8—C7—C6	119.6 (3)
C4—S1—C1	99.96 (11)	C8—C7—H7	120.2
C2—N1—C1	126.0 (2)	C7—C8—H8	119.9
C2—N1—C5	117.7 (2)	C7—C8—C9	120.2 (3)
C5—N1—C1	114.2 (2)	C9—C8—H8	119.9
S1—C1—H1	108.3	C8—C9—H9	119.8
N1—C1—S1	111.31 (17)	C8—C9—C10	120.4 (3)
N1—C1—H1	108.3	C10—C9—H9	119.8
N1—C1—C11	112.84 (19)	C5—C10—C9	119.1 (3)
C11—C1—S1	107.70 (15)	C5—C10—H10	120.5
C11—C1—H1	108.3	C9—C10—H10	120.5
S1—C4—H4A	109.9	C12—C11—C1	119.3 (2)
S1—C4—H4B	109.9	C12—C11—C16	119.7 (3)
H4A—C4—H4B	108.3	C16—C11—C1	121.0 (2)
C3—C4—S1	109.11 (19)	C11—C12—H12	120.1
C3—C4—H4A	109.9	C11—C12—C13	119.8 (3)
C3—C4—H4B	109.9	C13—C12—H12	120.1
C4—C3—H3A	107.6	C12—C13—H13	119.9
C4—C3—H3B	107.6	C14—C13—C12	120.2 (3)
C4—C3—C2	118.7 (2)	C14—C13—H13	119.9
H3A—C3—H3B	107.1	C13—C14—H14	120.0
C2—C3—H3A	107.6	C15—C14—C13	119.9 (3)
C2—C3—H3B	107.6	C15—C14—H14	120.0
O3—C2—N1	120.7 (2)	C14—C15—H15	119.7
O3—C2—C3	117.7 (2)	C14—C15—C16	120.7 (3)
N1—C2—C3	121.5 (2)	C16—C15—H15	119.7
C6—C5—N1	118.8 (2)	C11—C16—H16	120.2
C10—C5—N1	120.3 (2)	C15—C16—C11	119.6 (3)
C10—C5—C6	120.9 (2)	C15—C16—H16	120.2
			
S1—C1—C11—C12	−111.1 (2)	C4—C3—C2—O3	−167.2 (2)
S1—C1—C11—C16	68.1 (2)	C4—C3—C2—N1	15.3 (4)
S1—C4—C3—C2	−45.9 (3)	C2—N1—C1—S1	29.3 (3)
O1—S1—C1—N1	−168.32 (16)	C2—N1—C1—C11	150.5 (2)
O1—S1—C1—C11	67.50 (19)	C2—N1—C5—C6	96.5 (3)
O1—S1—C4—C3	172.01 (17)	C2—N1—C5—C10	−86.0 (3)
O2—S1—C1—N1	62.00 (19)	C5—N1—C1—S1	−167.57 (16)
O2—S1—C1—C11	−62.2 (2)	C5—N1—C1—C11	−46.3 (3)
O2—S1—C4—C3	−55.4 (2)	C5—N1—C2—O3	13.2 (3)
N1—C1—C11—C12	125.6 (2)	C5—N1—C2—C3	−169.4 (2)
N1—C1—C11—C16	−55.2 (3)	C5—C6—C7—C8	0.0 (5)
N1—C5—C6—C7	177.1 (3)	C6—C5—C10—C9	0.3 (5)
N1—C5—C10—C9	−177.2 (3)	C6—C7—C8—C9	0.7 (6)
C1—S1—C4—C3	60.16 (19)	C7—C8—C9—C10	−0.9 (6)
C1—N1—C2—O3	175.8 (2)	C8—C9—C10—C5	0.4 (6)
C1—N1—C2—C3	−6.8 (3)	C10—C5—C6—C7	−0.5 (4)
C1—N1—C5—C6	−68.1 (3)	C11—C12—C13—C14	−0.4 (4)
C1—N1—C5—C10	109.4 (3)	C12—C11—C16—C15	1.1 (4)
C1—C11—C12—C13	178.9 (2)	C12—C13—C14—C15	0.4 (4)
C1—C11—C16—C15	−178.1 (2)	C13—C14—C15—C16	0.4 (4)
C4—S1—C1—N1	−52.56 (18)	C14—C15—C16—C11	−1.2 (4)
C4—S1—C1—C11	−176.73 (18)	C16—C11—C12—C13	−0.3 (4)

### rac-2,3-Diphenyl-2,3,5,6-tetrahydro-4H-1,3-thiazine-1,1,4-trione (1) Hydrogen-bond geometry (Å, º)

**Table:**

*D*—H···*A*	*D*—H	H···*A*	*D*···*A*	*D*—H···*A*
C12—H12···O1^i^	0.95	2.56	3.454 (4)	157

Symmetry code: (i) −*x*+1, −*y*+1, −*z*+1.

### N-[(2S,5R)-1,1,4-Trioxo-2,3-diphenyl-1,3-thiazinan-5-yl]acetamide (2) Crystal data

**Table:**

C_18_H_18_N_2_O_4_S	*D*_x_ = 1.419 Mg m^−^^3^
*M**_r_* = 358.40	Mo *K*α radiation, λ = 0.71073 Å
Orthorhombic, *P*2_1_2_1_2_1_	Cell parameters from 4224 reflections
*a* = 5.5230 (4) Å	θ = 2.4–25.0°
*b* = 10.6857 (9) Å	µ = 0.22 mm^−^^1^
*c* = 28.430 (2) Å	*T* = 298 K
*V* = 1677.8 (2) Å^3^	Rod, colorless
*Z* = 4	0.22 × 0.06 × 0.06 mm
*F*(000) = 752	

### N-[(2S,5R)-1,1,4-Trioxo-2,3-diphenyl-1,3-thiazinan-5-yl]acetamide (2) Data collection

**Table:**

Bruker SMART CCD area detector diffractometer	3460 reflections with *I* > 2σ(*I*)
phi and ω scans	*R*_int_ = 0.035
Absorption correction: multi-scan (SADABS; Krause *et al.*, 2015)	θ_max_ = 28.3°, θ_min_ = 1.4°
*T*_min_ = 0.656, *T*_max_ = 0.900	*h* = −7→5
13301 measured reflections	*k* = −12→14
4037 independent reflections	*l* = −36→37

### N-[(2S,5R)-1,1,4-Trioxo-2,3-diphenyl-1,3-thiazinan-5-yl]acetamide (2) Refinement

**Table:**

Refinement on *F*^2^	Hydrogen site location: mixed
Least-squares matrix: full	H atoms treated by a mixture of independent and constrained refinement
*R*[*F*^2^ > 2σ(*F*^2^)] = 0.042	*w* = 1/[σ^2^(*F*_o_^2^) + (0.0564*P*)^2^ + 0.035*P*] where *P* = (*F*_o_^2^ + 2*F*_c_^2^)/3
*wR*(*F*^2^) = 0.103	(Δ/σ)_max_ < 0.001
*S* = 1.04	Δρ_max_ = 0.24 e Å^−^^3^
4037 reflections	Δρ_min_ = −0.14 e Å^−^^3^
231 parameters	Absolute structure: Flack *x* determined using 1213 quotients [(*I*^+^)-(*I*^-^)]/[(*I*^+^)+(*I*^-^)] (Parsons *et al.*, 2013)
0 restraints	Absolute structure parameter: 0.07 (4)
Primary atom site location: structure-invariant direct methods	

### N-[(2S,5R)-1,1,4-Trioxo-2,3-diphenyl-1,3-thiazinan-5-yl]acetamide (2) Special details

**Table:**

Experimental. The data collection nominally covered a full sphere of reciprocal space by a combination of 4 sets of ω scans each set at different φ and/or 2θ angles and each scan (10 s exposure) covering -0.300° degrees in ω. The crystal to detector distance was 5.82 cm.
Geometry. All esds (except the esd in the dihedral angle between two l.s. planes) are estimated using the full covariance matrix. The cell esds are taken into account individually in the estimation of esds in distances, angles and torsion angles; correlations between esds in cell parameters are only used when they are defined by crystal symmetry. An approximate (isotropic) treatment of cell esds is used for estimating esds involving l.s. planes.

### N-[(2S,5R)-1,1,4-Trioxo-2,3-diphenyl-1,3-thiazinan-5-yl]acetamide (2) Fractional atomic coordinates and isotropic or equivalent isotropic displacement parameters (Å^2^)

**Table:**

	*x*	*y*	*z*	*U*_iso_*/*U*_eq_	
S1	0.29097 (13)	0.61561 (6)	0.04750 (2)	0.04039 (18)	
O1	0.2013 (5)	0.5960 (2)	0.00084 (6)	0.0665 (7)	
O2	0.5430 (4)	0.5943 (2)	0.05600 (8)	0.0655 (6)	
O3	0.0948 (4)	0.72050 (18)	0.18769 (6)	0.0575 (6)	
O4	−0.3983 (4)	0.8940 (2)	0.13514 (8)	0.0659 (6)	
N1	0.1594 (4)	0.56459 (18)	0.13538 (6)	0.0394 (5)	
N2	0.0062 (5)	0.8977 (2)	0.12438 (9)	0.0485 (6)	
C1	0.1124 (4)	0.5198 (2)	0.08754 (7)	0.0333 (5)	
H1	−0.058432	0.536713	0.080716	0.040*	
C2	0.0930 (5)	0.6841 (2)	0.14753 (8)	0.0409 (6)	
C3	0.0251 (5)	0.7713 (2)	0.10656 (8)	0.0398 (6)	
H3	−0.132274	0.745690	0.093867	0.048*	
C4	0.2134 (6)	0.7692 (2)	0.06686 (8)	0.0424 (6)	
H4A	0.359008	0.811013	0.077660	0.051*	
H4B	0.150687	0.816206	0.040330	0.051*	
C11	0.1560 (4)	0.3832 (2)	0.07738 (7)	0.0350 (5)	
C16	0.3688 (5)	0.3203 (3)	0.08898 (9)	0.0448 (6)	
H16	0.493371	0.362002	0.104412	0.054*	
C15	0.3932 (6)	0.1953 (3)	0.07738 (10)	0.0559 (8)	
H15	0.533040	0.152566	0.085968	0.067*	
C14	0.2132 (7)	0.1331 (3)	0.05326 (10)	0.0596 (9)	
H14	0.232717	0.049213	0.045356	0.072*	
C13	0.0065 (7)	0.1949 (3)	0.04104 (10)	0.0565 (8)	
H13	−0.114479	0.153289	0.024516	0.068*	
C12	−0.0235 (5)	0.3194 (2)	0.05317 (8)	0.0434 (6)	
H12	−0.165681	0.360621	0.044990	0.052*	
C5	0.2702 (5)	0.4889 (2)	0.17177 (7)	0.0393 (6)	
C6	0.1536 (6)	0.3843 (3)	0.18873 (9)	0.0535 (8)	
H6	0.004073	0.360301	0.176670	0.064*	
C7	0.2639 (10)	0.3152 (3)	0.22429 (10)	0.0815 (13)	
H7	0.188747	0.243530	0.235747	0.098*	
C8	0.4806 (12)	0.3516 (5)	0.24243 (12)	0.0976 (17)	
H8	0.553113	0.304469	0.266063	0.117*	
C9	0.5929 (8)	0.4575 (5)	0.22597 (12)	0.0839 (13)	
H9	0.738587	0.483351	0.239181	0.101*	
C10	0.4902 (6)	0.5261 (3)	0.18983 (10)	0.0560 (8)	
H10	0.568552	0.596319	0.177902	0.067*	
C17	−0.2027 (7)	0.9434 (3)	0.14185 (9)	0.0490 (7)	
C18	−0.1753 (8)	1.0631 (3)	0.16996 (11)	0.0678 (10)	
H18A	−0.330040	1.103268	0.172852	0.102*	
H18B	−0.064566	1.118103	0.154150	0.102*	
H18C	−0.113853	1.043699	0.200700	0.102*	
H2	0.139 (5)	0.924 (3)	0.1348 (9)	0.042 (8)*	

### N-[(2S,5R)-1,1,4-Trioxo-2,3-diphenyl-1,3-thiazinan-5-yl]acetamide (2) Atomic displacement parameters (Å^2^)

**Table:**

	*U*^11^	*U*^22^	*U*^33^	*U*^12^	*U*^13^	*U*^23^
S1	0.0452 (4)	0.0358 (3)	0.0402 (3)	0.0022 (3)	0.0046 (3)	0.0016 (2)
O1	0.1092 (19)	0.0556 (12)	0.0347 (9)	−0.0001 (14)	0.0030 (11)	−0.0010 (8)
O2	0.0404 (11)	0.0512 (13)	0.1048 (17)	0.0035 (10)	0.0137 (12)	0.0130 (11)
O3	0.0835 (16)	0.0502 (12)	0.0387 (9)	0.0055 (11)	0.0013 (10)	−0.0095 (8)
O4	0.0562 (14)	0.0671 (15)	0.0742 (14)	0.0093 (13)	−0.0051 (11)	−0.0223 (12)
N1	0.0536 (15)	0.0329 (10)	0.0317 (9)	0.0008 (10)	−0.0029 (9)	−0.0018 (8)
N2	0.0530 (16)	0.0315 (12)	0.0611 (14)	−0.0018 (12)	−0.0034 (12)	−0.0078 (10)
C1	0.0321 (12)	0.0353 (12)	0.0324 (10)	0.0017 (10)	−0.0035 (9)	−0.0027 (9)
C2	0.0461 (16)	0.0349 (13)	0.0415 (12)	−0.0033 (12)	0.0008 (12)	−0.0033 (10)
C3	0.0431 (15)	0.0303 (12)	0.0460 (13)	−0.0002 (11)	−0.0029 (11)	−0.0054 (10)
C4	0.0506 (15)	0.0347 (12)	0.0419 (12)	−0.0013 (13)	−0.0016 (12)	0.0019 (9)
C11	0.0406 (14)	0.0327 (11)	0.0317 (10)	−0.0004 (11)	0.0015 (9)	−0.0009 (9)
C16	0.0480 (16)	0.0434 (14)	0.0431 (13)	0.0080 (13)	−0.0010 (11)	0.0002 (11)
C15	0.072 (2)	0.0458 (16)	0.0498 (15)	0.0223 (16)	0.0092 (15)	0.0110 (12)
C14	0.095 (3)	0.0319 (13)	0.0516 (15)	−0.0005 (17)	0.0225 (18)	0.0014 (11)
C13	0.072 (2)	0.0451 (16)	0.0521 (15)	−0.0183 (17)	0.0068 (15)	−0.0108 (12)
C12	0.0463 (15)	0.0445 (15)	0.0394 (12)	−0.0049 (13)	0.0010 (12)	−0.0051 (11)
C5	0.0445 (15)	0.0420 (13)	0.0315 (10)	0.0043 (12)	−0.0011 (11)	−0.0019 (9)
C6	0.071 (2)	0.0473 (15)	0.0422 (13)	−0.0011 (16)	0.0077 (13)	0.0033 (12)
C7	0.138 (4)	0.060 (2)	0.0462 (16)	0.015 (3)	0.012 (2)	0.0154 (14)
C8	0.147 (5)	0.100 (4)	0.0458 (18)	0.058 (3)	−0.020 (2)	0.0019 (19)
C9	0.073 (3)	0.118 (4)	0.060 (2)	0.039 (3)	−0.0289 (19)	−0.029 (2)
C10	0.0483 (18)	0.071 (2)	0.0483 (14)	0.0058 (16)	−0.0050 (13)	−0.0119 (14)
C17	0.065 (2)	0.0384 (14)	0.0435 (13)	0.0077 (15)	−0.0061 (15)	−0.0038 (10)
C18	0.095 (3)	0.0437 (16)	0.0649 (18)	0.0125 (19)	−0.0055 (19)	−0.0156 (13)

### N-[(2S,5R)-1,1,4-Trioxo-2,3-diphenyl-1,3-thiazinan-5-yl]acetamide (2) Geometric parameters (Å, º)

**Table:**

S1—O1	1.431 (2)	C15—H15	0.9300
S1—O2	1.431 (2)	C15—C14	1.378 (5)
S1—C1	1.821 (2)	C14—H14	0.9300
S1—C4	1.783 (3)	C14—C13	1.364 (5)
O3—C2	1.206 (3)	C13—H13	0.9300
O4—C17	1.218 (4)	C13—C12	1.384 (4)
N1—C1	1.465 (3)	C12—H12	0.9300
N1—C2	1.373 (3)	C5—C6	1.377 (4)
N1—C5	1.449 (3)	C5—C10	1.378 (4)
N2—C3	1.446 (3)	C6—H6	0.9300
N2—C17	1.348 (4)	C6—C7	1.393 (5)
N2—H2	0.84 (3)	C7—H7	0.9300
C1—H1	0.9800	C7—C8	1.360 (7)
C1—C11	1.507 (3)	C8—H8	0.9300
C2—C3	1.538 (3)	C8—C9	1.373 (6)
C3—H3	0.9800	C9—H9	0.9300
C3—C4	1.535 (4)	C9—C10	1.384 (5)
C4—H4A	0.9700	C10—H10	0.9300
C4—H4B	0.9700	C17—C18	1.515 (4)
C11—C16	1.394 (4)	C18—H18A	0.9600
C11—C12	1.386 (3)	C18—H18B	0.9600
C16—H16	0.9300	C18—H18C	0.9600
C16—C15	1.382 (4)		
			
O1—S1—C1	108.03 (13)	C16—C15—H15	119.5
O1—S1—C4	109.74 (13)	C14—C15—C16	121.0 (3)
O2—S1—O1	118.01 (15)	C14—C15—H15	119.5
O2—S1—C1	109.38 (12)	C15—C14—H14	120.1
O2—S1—C4	109.15 (14)	C13—C14—C15	119.8 (3)
C4—S1—C1	101.20 (11)	C13—C14—H14	120.1
C2—N1—C1	119.34 (19)	C14—C13—H13	119.9
C2—N1—C5	116.91 (18)	C14—C13—C12	120.2 (3)
C5—N1—C1	123.75 (19)	C12—C13—H13	119.9
C3—N2—H2	112 (2)	C11—C12—H12	119.6
C17—N2—C3	122.0 (3)	C13—C12—C11	120.7 (3)
C17—N2—H2	120 (2)	C13—C12—H12	119.6
S1—C1—H1	107.1	C6—C5—N1	120.4 (2)
N1—C1—S1	107.50 (16)	C10—C5—N1	118.5 (3)
N1—C1—H1	107.1	C10—C5—C6	121.1 (3)
N1—C1—C11	117.76 (19)	C5—C6—H6	120.7
C11—C1—S1	109.76 (16)	C5—C6—C7	118.7 (3)
C11—C1—H1	107.1	C7—C6—H6	120.7
O3—C2—N1	122.4 (2)	C6—C7—H7	119.7
O3—C2—C3	121.6 (2)	C8—C7—C6	120.6 (4)
N1—C2—C3	115.99 (19)	C8—C7—H7	119.7
N2—C3—C2	108.5 (2)	C7—C8—H8	119.9
N2—C3—H3	109.0	C7—C8—C9	120.2 (4)
N2—C3—C4	108.7 (2)	C9—C8—H8	119.9
C2—C3—H3	109.0	C8—C9—H9	119.8
C4—C3—C2	112.5 (2)	C8—C9—C10	120.3 (4)
C4—C3—H3	109.0	C10—C9—H9	119.8
S1—C4—H4A	108.8	C5—C10—C9	119.0 (4)
S1—C4—H4B	108.8	C5—C10—H10	120.5
C3—C4—S1	113.79 (17)	C9—C10—H10	120.5
C3—C4—H4A	108.8	O4—C17—N2	123.0 (2)
C3—C4—H4B	108.8	O4—C17—C18	122.5 (3)
H4A—C4—H4B	107.7	N2—C17—C18	114.5 (3)
C16—C11—C1	123.8 (2)	C17—C18—H18A	109.5
C12—C11—C1	117.2 (2)	C17—C18—H18B	109.5
C12—C11—C16	118.9 (2)	C17—C18—H18C	109.5
C11—C16—H16	120.3	H18A—C18—H18B	109.5
C15—C16—C11	119.5 (3)	H18A—C18—H18C	109.5
C15—C16—H16	120.3	H18B—C18—H18C	109.5
			
S1—C1—C11—C16	73.2 (2)	C2—N1—C5—C6	114.0 (3)
S1—C1—C11—C12	−103.8 (2)	C2—N1—C5—C10	−64.0 (3)
O1—S1—C1—N1	−165.13 (17)	C2—C3—C4—S1	50.6 (3)
O1—S1—C1—C11	65.65 (19)	C3—N2—C17—O4	−15.9 (4)
O1—S1—C4—C3	111.3 (2)	C3—N2—C17—C18	165.1 (2)
O2—S1—C1—N1	65.22 (19)	C4—S1—C1—N1	−49.88 (19)
O2—S1—C1—C11	−64.00 (19)	C4—S1—C1—C11	−179.10 (17)
O2—S1—C4—C3	−117.9 (2)	C11—C16—C15—C14	2.0 (4)
O3—C2—C3—N2	8.7 (4)	C16—C11—C12—C13	0.6 (4)
O3—C2—C3—C4	129.0 (3)	C16—C15—C14—C13	−0.7 (4)
N1—C1—C11—C16	−50.2 (3)	C15—C14—C13—C12	−0.6 (4)
N1—C1—C11—C12	132.9 (2)	C14—C13—C12—C11	0.7 (4)
N1—C2—C3—N2	−169.1 (2)	C12—C11—C16—C15	−1.9 (4)
N1—C2—C3—C4	−48.8 (3)	C5—N1—C1—S1	−117.4 (2)
N1—C5—C6—C7	−178.9 (3)	C5—N1—C1—C11	7.2 (3)
N1—C5—C10—C9	177.3 (3)	C5—N1—C2—O3	−9.9 (4)
N2—C3—C4—S1	170.82 (19)	C5—N1—C2—C3	167.9 (2)
C1—S1—C4—C3	−2.7 (2)	C5—C6—C7—C8	1.1 (5)
C1—N1—C2—O3	169.2 (3)	C6—C5—C10—C9	−0.8 (4)
C1—N1—C2—C3	−13.1 (4)	C6—C7—C8—C9	0.3 (6)
C1—N1—C5—C6	−64.9 (3)	C7—C8—C9—C10	−2.0 (6)
C1—N1—C5—C10	117.0 (3)	C8—C9—C10—C5	2.2 (5)
C1—C11—C16—C15	−178.8 (2)	C10—C5—C6—C7	−0.9 (4)
C1—C11—C12—C13	177.7 (2)	C17—N2—C3—C2	−88.7 (3)
C2—N1—C1—S1	63.7 (3)	C17—N2—C3—C4	148.7 (3)
C2—N1—C1—C11	−171.8 (2)		

### N-[(2S,5R)-1,1,4-Trioxo-2,3-diphenyl-1,3-thiazinan-5-yl]acetamide (2) Hydrogen-bond geometry (Å, º)

**Table:**

*D*—H···*A*	*D*—H	H···*A*	*D*···*A*	*D*—H···*A*
C1—H1···O2^i^	0.98	2.39	3.365 (3)	173
C4—H4*A*···O4^ii^	0.97	2.29	3.185 (4)	153
C8—H8···O3^iii^	0.93	2.51	3.378 (5)	155

Symmetry codes: (i) *x*−1, *y*, *z*; (ii) *x*+1, *y*, *z*; (iii) −*x*+1, *y*−1/2, −*z*+1/2.
